# Crystal structures of 3,6-di­allyl­tetra­cyclo[6.3.0.0^4,11^.0^5,9^]undeca-2,7-dione and 1,7-di­allyl­penta­cyclo­[5.4.0.0^2,6^. 0^3,10^.0^5,9^]undecane-8,11-dione: allyl­ated caged compounds

**DOI:** 10.1107/S1600536814023149

**Published:** 2014-10-24

**Authors:** Sambasivarao Kotha, Vital Seema, Deepak Deodhar, Mobin Shaikh

**Affiliations:** a Department of Chemistry, Indian Institute of Technology–Bombay, Powai, Mumbai 400 076, India

**Keywords:** Crystal structure, caged compounds, penta­cyclo [5.4.0.0^2,6^.0^3,10^.0^5,9^]undecane (PCUD), ring-closing metathesis (RCM), Diels–Alder reaction, [2 + 2] cyclo­additions, crystal structure

## Abstract

The title compounds, C_17_H_20_O_2_ (1) and C_17_H_18_O_2_ (2), are allyl­ated caged compounds. In (1), the carbon atoms bearing the allyl groups are far apart [2.9417 (17) Å], hence the expected ring-closing metathesis (RCM) protocol failed to give a ring-closing product. When these carbon atoms are connected by a C—C bond as in (2), the distance between them is much smaller [1.611 (3) Å] and consequently the RCM process was successful. The caged carbon skeleton of (1) can be described as a fusion of four five-membered rings and one six-membered ring. All four five-membered rings exhibit envelope conformations. The structure of compound (2) consists of four five-membered rings, of which two are cyclo­penta­none rings bonded at the 2, 4 and 5 positions and linked at the 3-carbons by a methyl­ene bridge. It also consists of one four-membered and two six-membered rings. All four five-membered rings adopt envelope conformations. In the crystal of (1), mol­ecules are linked *via* C—H⋯O hydrogen bonds, forming sheets lying parallel to (010). In the crystal of (2), mol­ecules are linked *via* C—H⋯O hydrogen bonds forming chains along [100].

## Abstract 2

The crystal structures of two allyl­ated caged mol­ecules and the correlation of bond distances and feasibility of ring-closing metathesis is discussed.

## Chemical context   

Caged mol­ecules are much sought after chemical entities due to their diverse applications such as high-energy materials, drug inter­mediates and starting materials for complex natural products (Marchand, 1989*a* ▶,*b* ▶; Mehta & Srikrishna, 1997 ▶). The intricacies involved in the structural frame of caged mol­ecules, such as deformation of ideal C—C bond angle and other unusual structural features, make them challenging synthetic targets (Olah, 1990 ▶; Osawa & Yonemitsu, 1992 ▶). Caged mol­ecules are strained due to the rigid geometrical features and they exhibit inter­esting properties (Von *et al.*, 1986 ▶): the high negative heat of combustion and elevated positive heat of formation for caged compounds reveal the strain involved in their mol­ecular architecture.
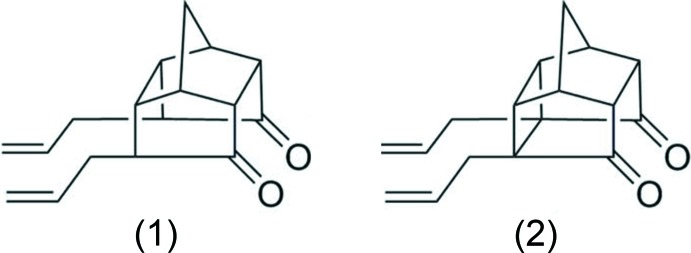


In connection with our inter­est in designing new varieties of caged compounds, we have synthesized several functionalized derivatives of penta­cyclo [5.4.0.0^2,6^.0^3,10^.0^5,9^]undecane (PCUD) systems (Kotha & Dipak, 2006 ▶; Kotha *et al.*, 2010 ▶). Herein, we report on the crystal structures of the title compounds, (1) and (2). These compounds, and their reactions mentioned in this article, are known in the literature (Kotha *et al.*, 1999 ▶, 2006 ▶) but their crystal structures have not previously been reported.

**Figure 1:**
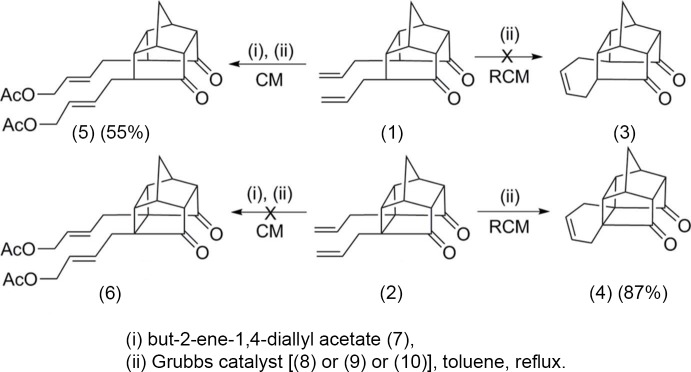
Synthesis of cage systems (1) and (2).

**Figure 2:**
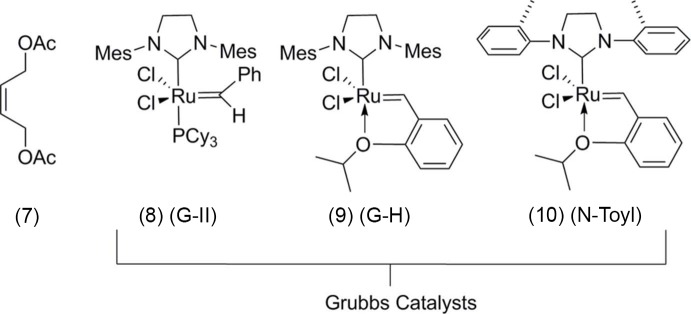
Various Grubbs catalysts used for ring-closing metathesis (RCM).

When diallyl tetra­cyclic dione (1) was subjected to ring-closing metathesis (RCM), the expected ring-closing product (3) was not obtained, Fig. 1 ▶. Whereas, compound (2) successfully underwent RCM to yield the desired ring-closing product (4), see Fig. 1 ▶. Further, when compound (1) was subjected to cross metathesis (CM) with but-2-ene-1,4-diallyl acetate (7) in the presence of Grubbs catalyst (Fig. 2 ▶), the di­acetate (5) was formed in 55% yield. Under similar reaction conditions, the penta­cyclic dione (2) did not deliver the cross-coupled product (6), but instead the RCM product (4) was formed, see Fig. 1 ▶. To gain insight about these observations, the crystal structure determinations of compounds (1) and (2) were undertaken.

## Structural commentary   

**Figure 3:**
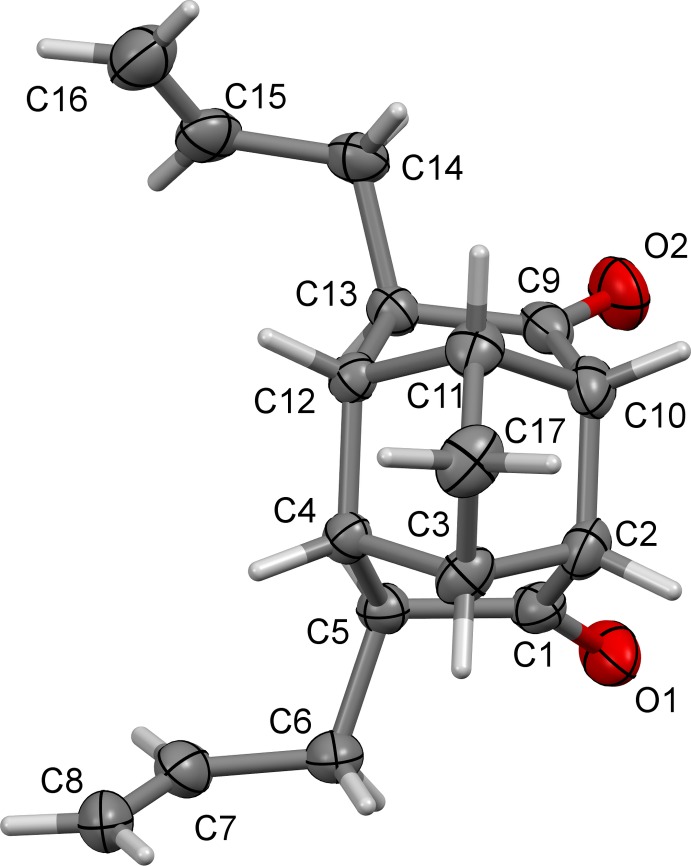
A view of the mol­ecular structure of compound (1), with atom labelling. Displacement ellipsoids are drawn at the 50% probability level.

The caged carbon skeleton of (1), Fig. 3 ▶, can be described as a fusion of four five-membered rings and one six-membered ring, the latter having a boat conformation. All four five-membered rings exhibit envelope conformations, with atoms C3, twice C17, and C11 as the flap atoms of the various rings. Compound (1) is symmetrically substituted with two allyl groups at atoms C5 and C10. The few crystal structures of PCUD compounds that are recorded in Cambridge Structural Database (Groom & Allen, 2014 ▶) show no bridging route through the substituents that link the C-atoms [*e.g.* C1 to C9, Fig. 3 ▶]. These compounds are substituted at C1 and/or C9 so that these mol­ecules form the open mouth of the cage. The tetra­cyclic compound (1) shows symmetrical substitution with keto moieties at atoms C1 and C9.

The C—C strained bond angles in (1) vary from 95.31 (10) to 125.21 (14)°, deviating from the ideal tetra­hedral angle of 109.5°. Previous studies showed that PCUD caged compounds normally display C—C bond lengths which deviate from expected value of 1.54 Å (Bott *et al.*, 1998 ▶; Flippen-Anderson *et al.*, 1991 ▶; Linden *et al.*, 2005 ▶; Kruger *et al.*, 2005 ▶). The structure of (1) also exhibits unusual C*sp* ^3^—C*sp* ^3^ single-bond lengths ranging from 1.5092 (19) Å to 1.5935 (19) Å. The bond C2—C10, which is parallel and immediately adjacent to C1—C9 axis, was found to be longer, with a value of 1.5935 (19) Å. The increase in bond length can be the result of stretching strain commenced by the open mouth of the cage formed by carbonyls bearing carbon atoms, *i.e.* C1 and C9. Similar observations were made in compound (2), *i.e.* 1.597 (4) Å for C5—C10.

**Figure 4:**
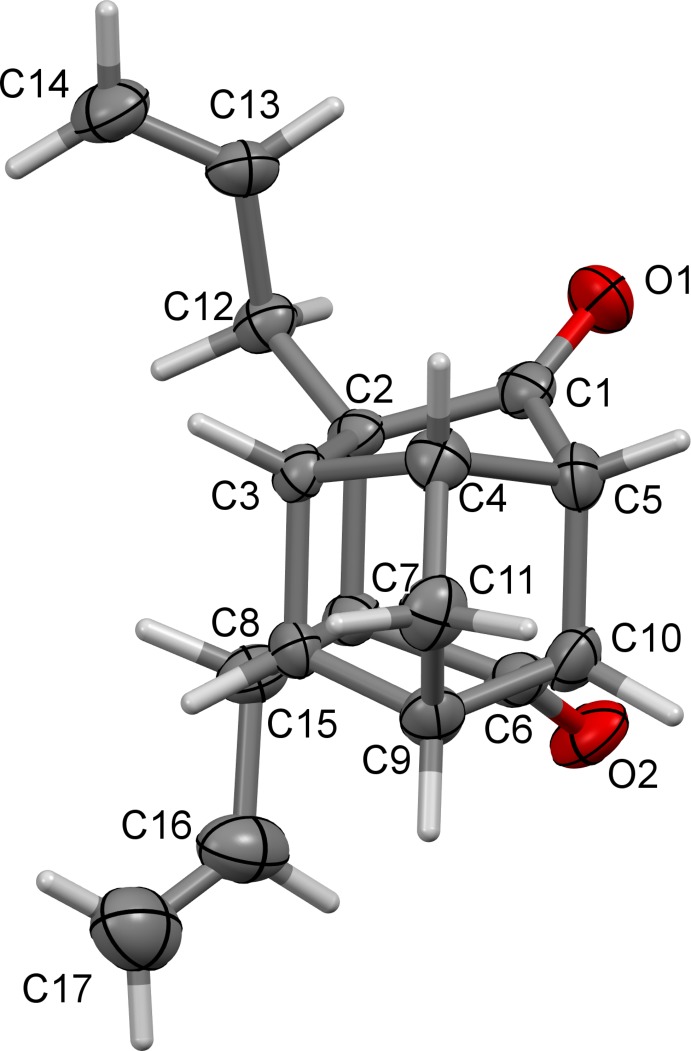
A view of the mol­ecular structure of compound (2), with atom labelling. Displacement ellipsoids are drawn at the 50% probability level.

The structure of compound (2), Fig. 4 ▶, consists of four five-membered rings, of which two are cyclo­penta­none rings, bonded at the 2, 4 and 5 positions and linked at the 3-carbons by a methyl­ene bridge. It also consists of one four-membered and two six-membered rings, the latter both having a boat conformation. All four five-membered rings adopt envelope conformations, with atoms C5, twice C11, and C10 as the flaps atoms of the various rings. Bonds C4—C11 and C7—C15, corresponding to 1.522 (4) and 1.522 (3) Å, respectively, are the shortest. The longest C—C bonds *i.e.* C2—C7 [1.611 (3) Å] and C5—C10 [1.597 (4) Å], along with C2—C3, C3—C4 and C7—C8 exceed the expected bond-length value of 1.54 Å. The bonds involving the bridge-head atom C11 are shorter than expected; C9—C11 and C4—C11 being 1.523 (4) and 1.522 (4) Å, respectively. The tetra­hedral bond angle C8—C7—C2 is the most strained with the smallest angle of 88.77 (17)° and the C15—C7—C8 bond angle of 119.6 (2)° is the largest one, again showing considerable deviation from the standard value of 109.5°.

It was anti­cipated that the two allyl groups present in (1) would undergo RCM to generate a new penta­cyclic system (3) (Fig. 1 ▶). However, it was observed that even under forcing reaction conditions, (1) did not generate the expected RCM product, whereas compound (2) underwent an RCM sequence smoothly to give (4) in good yield (Fig. 1 ▶). It was found that the allyl-bearing carbon atoms in tetra­cyclic system (1) are too far apart [C5—C13 = 2.9417 (17) Å] and we believe that due to this reason, the RCM protocol failed. When these carbon atoms are bonded, the distance between them was found to be smaller. Thus in (2), the distance between the bonded atoms C2—C7 is 1.611 (3) Å.

During CM, Fig. 1 ▶, dione (2) was reacted with but-2-ene-1,4-diallyl acetate (7) to produce cross-coupling product (6). However, (2) failed to deliver the CM product, but under similar conditions, (1) successfully gave the di­acetate (5). In the present scenario, the distance between the allyl-bearing carbon atoms in (1) and (2) has been correlated to understand the reactivity pattern. When the distance between these carbon atoms is large as in the case of (1), the CM product is preferred over RCM, and when the distance is smaller, the RCM product is predominant over the CM product.

The conclusion is that, as the C5—C13 separation in (1) is large [2.9417 (17) Å], the carbon atoms bearing the allyl groups are far apart in this tetra­cyclic system, and the expected ring-closing metathesis (RCM) protocol failed to give the ring-closing product (3), Fig. 1 ▶. When these carbon atoms are connected by a C—C bond as in (2), the C2—C7 bond distance was found to be much smaller [1.611 (3) Å], and consequently the RCM process was successful giving the diallyl compound (4), Fig. 1 ▶.

## Supra­molecular features   

**Figure 5:**
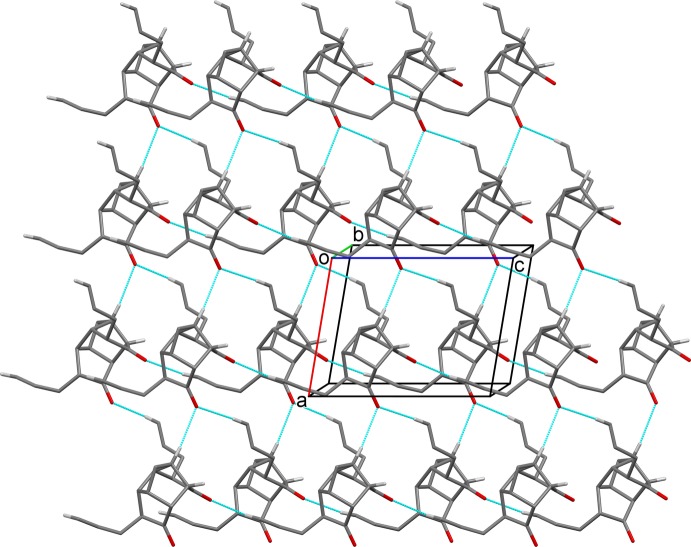
A view along the *b* axis of the crystal packing of compound (1). Hydrogen bonds are shown as dashed lines (see Table 1 ▶ for details; only the H atoms involved in these hydrogen bonds are shown).

In the crystal of (1), mol­ecules are linked *via* C—H⋯O hydrogen bonds, forming sheets lying parallel to (010); see Fig. 5 ▶ and Table 1 ▶.

**Figure 6:**
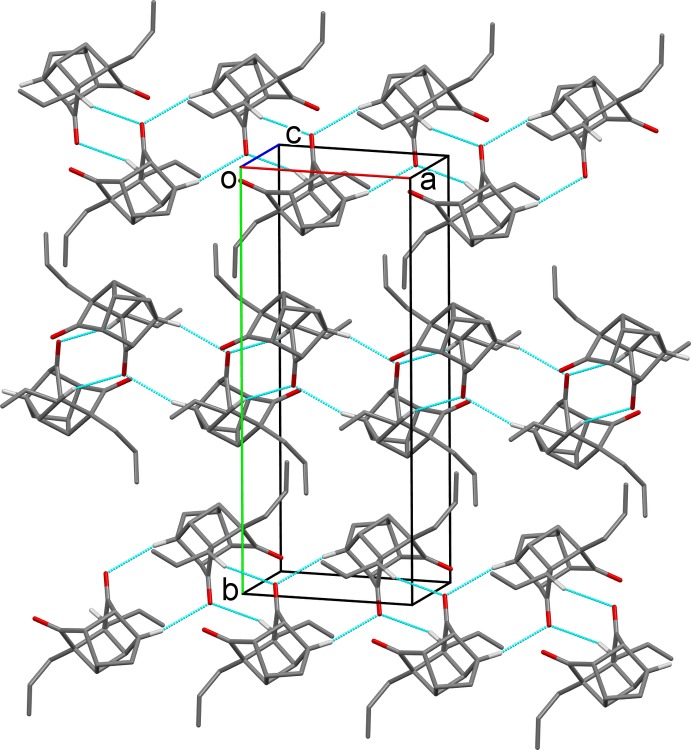
A view along the *c* axis of the crystal packing of compound (2). Hydrogen bonds are shown as dashed lines (see Table 2 ▶ for details; only the H atoms involved in these hydrogen bonds are shown).

In the crystal of (2), mol­ecules are linked *via* C—H⋯O hydrogen bonds, forming chains along [100]; see Fig. 6 ▶ and Table 2 ▶.

## Synthesis and crystallization   

Compounds (1) and (2) were prepared by the procedures reported in the literature (Kotha *et al.*, 1999 ▶ and Kotha *et al.*, 2006 ▶, respectively) and their melting points were compared with the reported values. In addition, their identity was confirmed by NMR spectroscopic data.

**Compound (1):** The crude compound (1) was obtained after reaction work-up and was purified using silica gel column chromatography (3% EtOAc/petroleum ether). Colourless crystals were isolated when the solvent was allowed to evaporate (m.p. 356.15–357.15 K; literature m.p. 357.15–358.15 K).

**Compound (2):** The crude compound (2) was obtained after reaction work-up and was purified using silica gel column chromatography (5% EtOAc/petroleum ether). Colourless crystals were isolated when the solvent was allowed to evaporate (m.p. 353.15–354.15 K; literature m.p. 353.15–354.15 K).

## Refinement   

Crystal data, data collection and structure refinement details of compounds (1) and (2) are summarized in the Table 3 ▶. For both the compounds all H atoms were placed in geometrically calculated positions and refined using a riding model, with C—H = 0.95–1.00 Å and with *U*_iso_(H) = 1.2*U*_eq_(C).

## Supplementary Material

Crystal structure: contains datablock(s) 1, 2, global. DOI: 10.1107/S1600536814023149/su5002sup1.cif

Structure factors: contains datablock(s) 1. DOI: 10.1107/S1600536814023149/su50021sup2.hkl

Structure factors: contains datablock(s) 2. DOI: 10.1107/S1600536814023149/su50022sup3.hkl

Supporting information file. DOI: 10.1107/S1600536814023149/su50022sup4.cml

CCDC references: 832290, 963826

Additional supporting information:  crystallographic information; 3D view; checkCIF report

## Figures and Tables

**Table 1:** Hydrogen-bond geometry (, ) for (1)

*D*H*A*	*D*H	H*A*	*D* *A*	*D*H*A*
C8H8*B*O2^i^	0.95	2.42	3.3532(18)	168
C11H11O1^ii^	1.00	2.49	3.4815(16)	173
C16H16*B*O1^iii^	0.95	2.51	3.455(2)	177

Symmetry codes: (i) 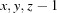; (ii) 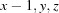; (iii) 
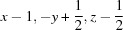
.

**Table 2:** Hydrogen-bond geometry (, ) for (2)

*D*H*A*	*D*H	H*A*	*D* *A*	*D*H*A*
C9H9O2^i^	1.00	2.44	3.412(3)	165
C15H15*B*O2^ii^	0.99	2.43	3.383(3)	160

Symmetry codes: (i) 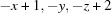; (ii) 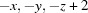.

**Table 3:** Experimental details

	(1)	(2)
Crystal data		
Chemical formula	C_17_H_20_O_2_	C_17_H_18_O_2_
*M*_r_	256.33	254.31
Crystal system, space group	Monoclinic, *P*2_1_/*c*	Monoclinic, *P*2_1_/*c*
Temperature (K)	150	150
*a*, *b*, *c* ()	7.8006(3), 17.9581(7), 10.1032(4)	8.7041(5), 18.3992(9), 9.0906(6)
()	99.664(4)	113.043(7)
*V* (^3^)	1395.21(9)	1339.69(13)
*Z*	4	4
Radiation type	Mo *K*	Mo *K*
(mm^1^)	0.08	0.08
Crystal size (mm)	0.29 0.25 0.21	0.32 0.28 0.23

Data collection		
Diffractometer	Oxford Diffraction Xcalibur-S	Oxford Diffraction Xcalibur-S
Absorption correction	Multi-scan (*CrysAlis RED*; Oxford Diffraction, 2006 ▶)	Multi-scan (*CrysAlis RED*; Oxford Diffraction, 2006 ▶)
*T*_min_, *T*_max_	0.978, 0.984	0.975, 0.982
No. of measured, independent and observed [*I* > 2(*I*)] reflections	9836, 2448, 1988	8644, 2356, 1625
*R*_int_	0.020	0.036
(sin /)_max_ (^1^)	0.595	0.595

Refinement		
*R*[*F* ^2^ > 2(*F* ^2^)], *wR*(*F* ^2^), *S*	0.035, 0.099, 1.06	0.059, 0.188, 1.10
No. of reflections	2448	2356
No. of parameters	172	172
H-atom treatment	H-atom parameters constrained	H-atom parameters constrained
_max_, _min_ (e ^3^)	0.20, 0.15	0.10, 0.34

Computer programs: *CrysAlis CCD* and *CrysAlis RED* (Oxford Diffraction, 2006 ▶), *SHELXS97* and *SHELXL97* (Sheldrick, 2008 ▶), *Mercury* (Macrae *et al.*, 2008 ▶), *PLATON* (Spek, 2009 ▶) and *publCIF* (Westrip, 2010 ▶).

## supplementary crystallographic information

### Crystal data

**Table:**

C_17_H_18_O_2_	*F*(000) = 544
*M**_r_* = 254.31	*D*_x_ = 1.261 Mg m^−^^3^
Monoclinic, *P*2_1_/*c*	Melting point = 354.15–353.15 K
Hall symbol: -P 2ybc	Mo *K*α radiation, λ = 0.71073 Å
*a* = 8.7041 (5) Å	Cell parameters from 4211 reflections
*b* = 18.3992 (9) Å	θ = 3.3–32.4°
*c* = 9.0906 (6) Å	µ = 0.08 mm^−^^1^
β = 113.043 (7)°	*T* = 150 K
*V* = 1339.69 (13) Å^3^	Block, colourless
*Z* = 4	0.32 × 0.28 × 0.23 mm

### Data collection

**Table:**

Oxford Diffraction Xcalibur-S diffractometer	2356 independent reflections
Radiation source: Enhance (Mo) X-ray Source	1625 reflections with *I* > 2σ(*I*)
Graphite monochromator	*R*_int_ = 0.036
Detector resolution: 15.9948 pixels mm^-1^	θ_max_ = 25.0°, θ_min_ = 3.3°
ω/θ scans	*h* = −9→10
Absorption correction: multi-scan (*CrysAlis RED*; Oxford Diffraction, 2006)	*k* = −21→21
*T*_min_ = 0.975, *T*_max_ = 0.982	*l* = −10→10
8644 measured reflections	

### Refinement

**Table:**

Refinement on *F*^2^	Primary atom site location: structure-invariant direct methods
Least-squares matrix: full	Secondary atom site location: difference Fourier map
*R*[*F*^2^ > 2σ(*F*^2^)] = 0.059	Hydrogen site location: inferred from neighbouring sites
*wR*(*F*^2^) = 0.188	H-atom parameters constrained
*S* = 1.10	*w* = 1/[σ^2^(*F*_o_^2^) + (0.1199*P*)^2^] where *P* = (*F*_o_^2^ + 2*F*_c_^2^)/3
2356 reflections	(Δ/σ)_max_ < 0.001
172 parameters	Δρ_max_ = 0.10 e Å^−^^3^
0 restraints	Δρ_min_ = −0.34 e Å^−^^3^

### Special details

**Table:**

Geometry. All e.s.d.'s (except the e.s.d. in the dihedral angle between two l.s. planes) are estimated using the full covariance matrix. The cell e.s.d.'s are taken into account individually in the estimation of e.s.d.'s in distances, angles and torsion angles; correlations between e.s.d.'s in cell parameters are only used when they are defined by crystal symmetry. An approximate (isotropic) treatment of cell e.s.d.'s is used for estimating e.s.d.'s involving l.s. planes.
Refinement. Refinement of *F*^2^ against ALL reflections. The weighted *R*-factor *wR* and goodness of fit *S* are based on *F*^2^, conventional *R*-factors *R* are based on *F*, with *F* set to zero for negative *F*^2^. The threshold expression of *F*^2^ > σ(*F*^2^) is used only for calculating *R*-factors(gt) *etc*. and is not relevant to the choice of reflections for refinement. *R*-factors based on *F*^2^ are statistically about twice as large as those based on *F*, and *R*- factors based on ALL data will be even larger.

### Fractional atomic coordinates and isotropic or equivalent isotropic displacement parameters (Å^2^)

**Table:**

	*x*	*y*	*z*	*U*_iso_*/*U*_eq_	
O1	−0.1367 (2)	0.06672 (11)	0.5971 (2)	0.0424 (6)	
O2	0.2051 (2)	−0.02964 (10)	0.9446 (2)	0.0432 (6)	
C1	−0.0014 (3)	0.09420 (13)	0.6688 (3)	0.0304 (6)	
C2	0.0464 (3)	0.14322 (12)	0.8151 (3)	0.0253 (6)	
C3	0.1827 (3)	0.19321 (13)	0.7979 (3)	0.0248 (6)	
H3	0.1701	0.2466	0.8099	0.030*	
C4	0.2176 (3)	0.16845 (14)	0.6509 (3)	0.0322 (6)	
H4	0.1684	0.2000	0.5541	0.039*	
C5	0.1520 (3)	0.08931 (14)	0.6303 (3)	0.0336 (7)	
H5	0.1310	0.0678	0.5231	0.040*	
C6	0.2263 (3)	0.03044 (14)	0.9011 (3)	0.0322 (6)	
C7	0.1904 (3)	0.10269 (13)	0.9618 (3)	0.0268 (6)	
C8	0.3217 (3)	0.15429 (13)	0.9379 (3)	0.0267 (6)	
H8	0.3899	0.1851	1.0313	0.032*	
C9	0.4181 (3)	0.11108 (14)	0.8554 (3)	0.0331 (7)	
H9	0.5334	0.0955	0.9266	0.040*	
C10	0.2941 (3)	0.04889 (14)	0.7757 (3)	0.0341 (7)	
H10	0.3454	0.0066	0.7425	0.041*	
C11	0.4068 (3)	0.16081 (15)	0.7176 (3)	0.0377 (7)	
H11A	0.4651	0.2077	0.7544	0.045*	
H11B	0.4470	0.1372	0.6414	0.045*	
C12	−0.1015 (3)	0.17601 (13)	0.8421 (3)	0.0287 (6)	
H12A	−0.1786	0.1367	0.8435	0.034*	
H12B	−0.0609	0.2005	0.9475	0.034*	
C13	−0.1941 (3)	0.22985 (15)	0.7137 (3)	0.0345 (7)	
H13	−0.2499	0.2115	0.6084	0.041*	
C14	−0.2046 (4)	0.29917 (16)	0.7340 (4)	0.0441 (8)	
H14A	−0.1508	0.3199	0.8374	0.053*	
H14B	−0.2662	0.3292	0.6456	0.053*	
C15	0.1757 (3)	0.10004 (14)	1.1231 (3)	0.0338 (7)	
H15A	0.1541	0.1495	1.1532	0.041*	
H15B	0.0803	0.0687	1.1153	0.041*	
C16	0.3326 (4)	0.0708 (2)	1.2495 (4)	0.0542 (9)	
H16	0.3445	0.0194	1.2551	0.065*	
C17	0.4476 (5)	0.1061 (2)	1.3466 (4)	0.0664 (11)	
H17A	0.4422	0.1577	1.3462	0.080*	
H17B	0.5419	0.0818	1.4219	0.080*	

### Atomic displacement parameters (Å^2^)

**Table:**

	*U*^11^	*U*^22^	*U*^33^	*U*^12^	*U*^13^	*U*^23^
O1	0.0307 (11)	0.0387 (12)	0.0478 (12)	−0.0057 (9)	0.0047 (9)	−0.0107 (9)
O2	0.0398 (12)	0.0226 (10)	0.0648 (14)	0.0051 (9)	0.0180 (10)	0.0092 (9)
C1	0.0301 (14)	0.0206 (13)	0.0343 (14)	0.0013 (11)	0.0059 (12)	0.0028 (10)
C2	0.0248 (13)	0.0201 (12)	0.0297 (13)	0.0025 (10)	0.0092 (11)	0.0032 (10)
C3	0.0277 (13)	0.0180 (12)	0.0290 (13)	−0.0009 (10)	0.0113 (11)	0.0000 (9)
C4	0.0360 (15)	0.0319 (14)	0.0305 (13)	0.0004 (12)	0.0149 (12)	0.0021 (11)
C5	0.0365 (15)	0.0324 (15)	0.0312 (14)	−0.0005 (12)	0.0125 (12)	−0.0092 (11)
C6	0.0226 (13)	0.0251 (14)	0.0426 (15)	0.0024 (11)	0.0058 (12)	0.0022 (11)
C7	0.0254 (13)	0.0222 (13)	0.0309 (13)	0.0027 (10)	0.0089 (11)	0.0022 (10)
C8	0.0254 (13)	0.0237 (13)	0.0285 (13)	−0.0019 (10)	0.0080 (11)	−0.0041 (10)
C9	0.0238 (13)	0.0363 (15)	0.0395 (15)	0.0017 (11)	0.0127 (12)	−0.0053 (12)
C10	0.0314 (15)	0.0274 (14)	0.0450 (16)	0.0048 (11)	0.0166 (13)	−0.0066 (11)
C11	0.0366 (16)	0.0394 (16)	0.0446 (16)	−0.0006 (13)	0.0239 (13)	−0.0019 (13)
C12	0.0260 (13)	0.0249 (13)	0.0359 (14)	0.0003 (11)	0.0127 (11)	0.0030 (11)
C13	0.0261 (14)	0.0387 (16)	0.0367 (14)	0.0062 (12)	0.0100 (12)	0.0040 (12)
C14	0.0397 (17)	0.0329 (16)	0.0550 (18)	0.0086 (13)	0.0136 (15)	0.0089 (13)
C15	0.0332 (14)	0.0334 (15)	0.0358 (14)	0.0057 (12)	0.0146 (12)	0.0085 (12)
C16	0.0457 (19)	0.078 (2)	0.0377 (17)	0.0160 (18)	0.0153 (15)	0.0077 (17)
C17	0.059 (2)	0.087 (3)	0.051 (2)	0.011 (2)	0.0193 (19)	−0.006 (2)

### Geometric parameters (Å, º)

**Table:**

O1—C1	1.211 (3)	C9—C11	1.523 (4)
O2—C6	1.212 (3)	C9—C10	1.545 (4)
C1—C5	1.510 (4)	C9—H9	1.0000
C1—C2	1.524 (4)	C10—H10	1.0000
C2—C12	1.526 (3)	C11—H11A	0.9900
C2—C3	1.557 (3)	C11—H11B	0.9900
C2—C7	1.611 (3)	C12—C13	1.502 (4)
C3—C8	1.546 (3)	C12—H12A	0.9900
C3—C4	1.550 (4)	C12—H12B	0.9900
C3—H3	1.0000	C13—C14	1.297 (4)
C4—C11	1.522 (4)	C13—H13	0.9500
C4—C5	1.549 (4)	C14—H14A	0.9500
C4—H4	1.0000	C14—H14B	0.9500
C5—C10	1.597 (4)	C15—C16	1.498 (4)
C5—H5	1.0000	C15—H15A	0.9900
C6—C10	1.513 (4)	C15—H15B	0.9900
C6—C7	1.518 (3)	C16—C17	1.229 (5)
C7—C15	1.522 (3)	C16—H16	0.9500
C7—C8	1.565 (4)	C17—H17A	0.9500
C8—C9	1.546 (4)	C17—H17B	0.9500
C8—H8	1.0000		
			
O1—C1—C5	127.7 (2)	C11—C9—C10	104.6 (2)
O1—C1—C2	126.4 (3)	C11—C9—C8	102.6 (2)
C5—C1—C2	105.9 (2)	C10—C9—C8	101.3 (2)
C1—C2—C12	114.5 (2)	C11—C9—H9	115.5
C1—C2—C3	102.7 (2)	C10—C9—H9	115.5
C12—C2—C3	120.4 (2)	C8—C9—H9	115.5
C1—C2—C7	107.84 (19)	C6—C10—C9	102.7 (2)
C12—C2—C7	118.8 (2)	C6—C10—C5	108.9 (2)
C3—C2—C7	88.83 (17)	C9—C10—C5	102.5 (2)
C8—C3—C4	102.6 (2)	C6—C10—H10	113.9
C8—C3—C2	91.47 (18)	C9—C10—H10	113.9
C4—C3—C2	109.09 (19)	C5—C10—H10	113.9
C8—C3—H3	116.7	C4—C11—C9	95.4 (2)
C4—C3—H3	116.7	C4—C11—H11A	112.7
C2—C3—H3	116.7	C9—C11—H11A	112.7
C11—C4—C5	104.6 (2)	C4—C11—H11B	112.7
C11—C4—C3	103.3 (2)	C9—C11—H11B	112.7
C5—C4—C3	101.1 (2)	H11A—C11—H11B	110.1
C11—C4—H4	115.3	C13—C12—C2	111.4 (2)
C5—C4—H4	115.3	C13—C12—H12A	109.3
C3—C4—H4	115.3	C2—C12—H12A	109.3
C1—C5—C4	103.5 (2)	C13—C12—H12B	109.3
C1—C5—C10	107.7 (2)	C2—C12—H12B	109.3
C4—C5—C10	102.0 (2)	H12A—C12—H12B	108.0
C1—C5—H5	114.1	C14—C13—C12	125.7 (3)
C4—C5—H5	114.1	C14—C13—H13	117.1
C10—C5—H5	114.1	C12—C13—H13	117.1
O2—C6—C10	127.2 (2)	C13—C14—H14A	120.0
O2—C6—C7	127.0 (2)	C13—C14—H14B	120.0
C10—C6—C7	105.9 (2)	H14A—C14—H14B	120.0
C6—C7—C15	115.4 (2)	C16—C15—C7	110.8 (2)
C6—C7—C8	102.5 (2)	C16—C15—H15A	109.5
C15—C7—C8	119.6 (2)	C7—C15—H15A	109.5
C6—C7—C2	108.00 (19)	C16—C15—H15B	109.5
C15—C7—C2	118.7 (2)	C7—C15—H15B	109.5
C8—C7—C2	88.77 (17)	H15A—C15—H15B	108.1
C3—C8—C9	103.7 (2)	C17—C16—C15	127.0 (4)
C3—C8—C7	90.93 (18)	C17—C16—H16	116.5
C9—C8—C7	108.6 (2)	C15—C16—H16	116.5
C3—C8—H8	116.7	C16—C17—H17A	120.0
C9—C8—H8	116.7	C16—C17—H17B	120.0
C7—C8—H8	116.7	H17A—C17—H17B	120.0
			
O1—C1—C2—C12	−17.7 (4)	C4—C3—C8—C9	−0.8 (2)
C5—C1—C2—C12	160.6 (2)	C2—C3—C8—C9	109.1 (2)
O1—C1—C2—C3	−150.1 (2)	C4—C3—C8—C7	−110.27 (19)
C5—C1—C2—C3	28.2 (2)	C2—C3—C8—C7	−0.32 (18)
O1—C1—C2—C7	117.0 (3)	C6—C7—C8—C3	108.48 (19)
C5—C1—C2—C7	−64.7 (2)	C15—C7—C8—C3	−122.4 (2)
C1—C2—C3—C8	−107.72 (19)	C2—C7—C8—C3	0.31 (17)
C12—C2—C3—C8	123.5 (2)	C6—C7—C8—C9	3.6 (2)
C7—C2—C3—C8	0.31 (18)	C15—C7—C8—C9	132.8 (2)
C1—C2—C3—C4	−3.8 (2)	C2—C7—C8—C9	−104.5 (2)
C12—C2—C3—C4	−132.6 (2)	C3—C8—C9—C11	34.2 (2)
C7—C2—C3—C4	104.2 (2)	C7—C8—C9—C11	129.9 (2)
C8—C3—C4—C11	−32.9 (2)	C3—C8—C9—C10	−73.7 (2)
C2—C3—C4—C11	−129.0 (2)	C7—C8—C9—C10	22.0 (2)
C8—C3—C4—C5	75.2 (2)	O2—C6—C10—C9	−135.8 (3)
C2—C3—C4—C5	−20.9 (2)	C7—C6—C10—C9	44.1 (2)
O1—C1—C5—C4	135.6 (3)	O2—C6—C10—C5	116.1 (3)
C2—C1—C5—C4	−42.6 (2)	C7—C6—C10—C5	−64.0 (2)
O1—C1—C5—C10	−116.8 (3)	C11—C9—C10—C6	−145.8 (2)
C2—C1—C5—C10	64.9 (2)	C8—C9—C10—C6	−39.4 (2)
C11—C4—C5—C1	145.1 (2)	C11—C9—C10—C5	−32.8 (2)
C3—C4—C5—C1	38.0 (2)	C8—C9—C10—C5	73.5 (2)
C11—C4—C5—C10	33.3 (2)	C1—C5—C10—C6	−0.5 (3)
C3—C4—C5—C10	−73.8 (2)	C4—C5—C10—C6	108.0 (2)
O2—C6—C7—C15	19.1 (4)	C1—C5—C10—C9	−108.8 (2)
C10—C6—C7—C15	−160.8 (2)	C4—C5—C10—C9	−0.3 (2)
O2—C6—C7—C8	150.9 (3)	C5—C4—C11—C9	−52.5 (2)
C10—C6—C7—C8	−29.1 (2)	C3—C4—C11—C9	53.0 (2)
O2—C6—C7—C2	−116.4 (3)	C10—C9—C11—C4	52.2 (2)
C10—C6—C7—C2	63.7 (2)	C8—C9—C11—C4	−53.2 (2)
C1—C2—C7—C6	0.0 (3)	C1—C2—C12—C13	−67.8 (3)
C12—C2—C7—C6	132.4 (2)	C3—C2—C12—C13	55.5 (3)
C3—C2—C7—C6	−103.0 (2)	C7—C2—C12—C13	162.7 (2)
C1—C2—C7—C15	−133.8 (2)	C2—C12—C13—C14	−114.1 (3)
C12—C2—C7—C15	−1.3 (3)	C6—C7—C15—C16	58.3 (3)
C3—C2—C7—C15	123.2 (2)	C8—C7—C15—C16	−64.7 (3)
C1—C2—C7—C8	102.7 (2)	C2—C7—C15—C16	−171.2 (2)
C12—C2—C7—C8	−124.9 (2)	C7—C15—C16—C17	98.4 (4)
C3—C2—C7—C8	−0.31 (17)		

### Hydrogen-bond geometry (Å, º)

**Table:**

*D*—H···*A*	*D*—H	H···*A*	*D*···*A*	*D*—H···*A*
C9—H9···O2^i^	1.00	2.44	3.412 (3)	165
C15—H15*B*···O2^ii^	0.99	2.43	3.383 (3)	160

Symmetry codes: (i) −*x*+1, −*y*, −*z*+2; (ii) −*x*, −*y*, −*z*+2.
